# Plastic pollution and antimicrobial resistance: an emerging link with major implications

**DOI:** 10.1128/aem.00973-26

**Published:** 2026-06-16

**Authors:** Zhi Mei, Erika A. Rodríguez, José L. Balcázar

**Affiliations:** 1 Catalan Institute for Water Research (ICRA-CERCA) 504638, Girona, Spain; Michigan State University, East Lansing, Michigan, USA

**Keywords:** antimicrobial resistance, microplastics, horizontal gene transfer

## Abstract

Plastic pollution and antimicrobial resistance are increasingly interconnected global threats. Micro- and nanoplastics create ecological hotspots that enhance microbial interactions and horizontal gene transfer, facilitating antimicrobial resistance dissemination. Here, we argue that the plastisphere acts as an evolutionary interface that reshapes microbial adaptation and resistome dynamics across ecosystems. Current antimicrobial resistance surveillance frameworks largely overlook the contribution of plastic pollution, highlighting the need to integrate plastisphere-mediated processes into One Health and environmental risk assessment strategies.

## COMMENTARY

Plastic pollution is pervasive throughout the biosphere, from soils and freshwater systems to the atmosphere and remote environments. Antimicrobial resistance is now recognized as one of the most pressing global health threats of the 21st century. These crises are often addressed as separate challenges, despite their deep interconnection. Micro- and nanoplastics, together with the microbial communities they host, form a critical interface linking environmental contamination to the evolution and dissemination of antimicrobial resistance. This connection indicates that plastic pollution functions not only as an environmental burden but also as a factor shaping microbial evolution.

**Fig 1:**
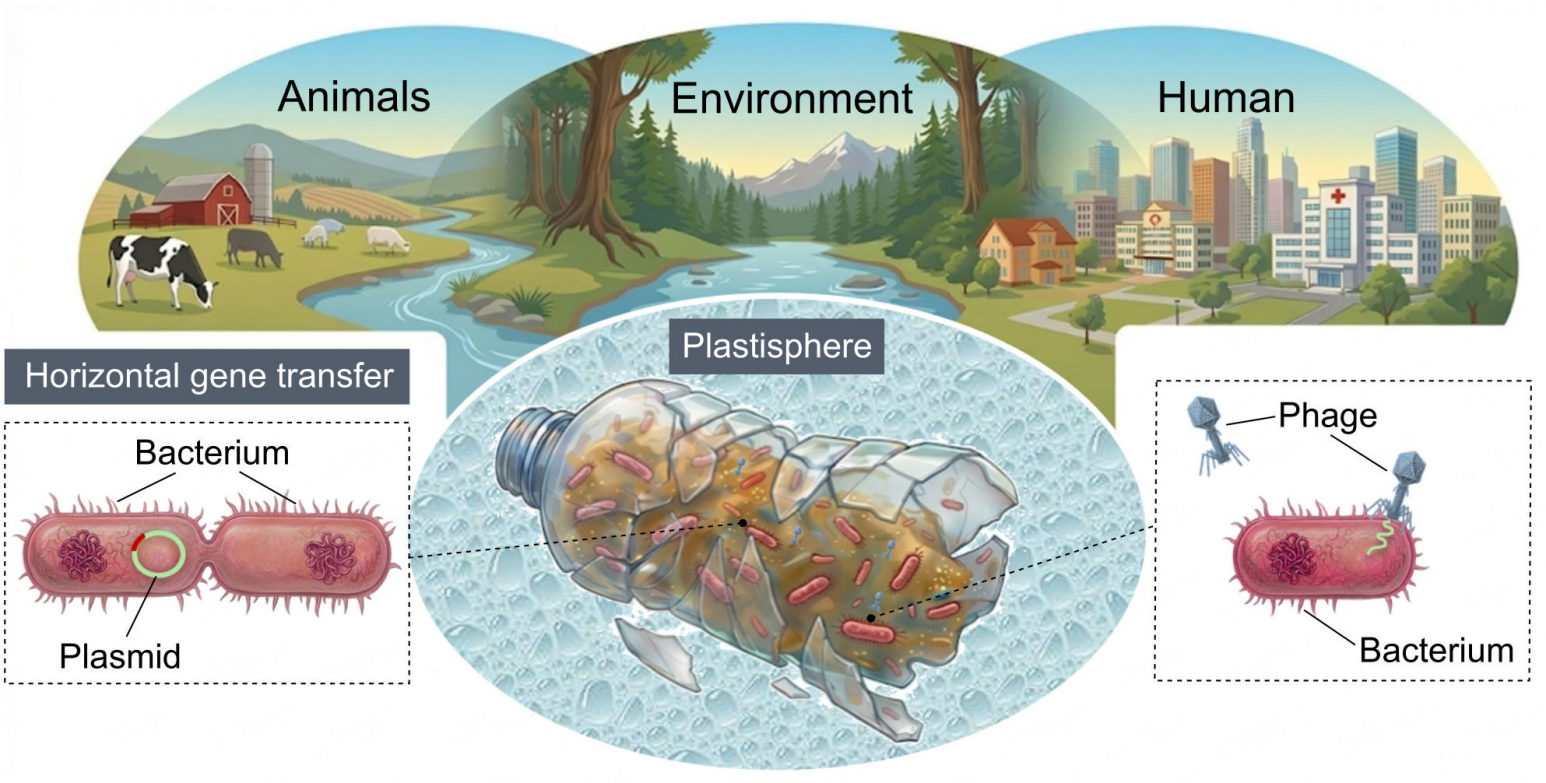
Plastisphere-mediated pathways linking plastic pollution to antimicrobial resistance across ecosystems. Micro- and nanoplastics support biofilm formation and create hotspots for contaminant accumulation and horizontal gene transfer. Interactions among bacteria, plasmids, and phages within the plastisphere facilitate the dissemination of antimicrobial resistance genes across interconnected human, animal, and environmental systems.

Plastic pollution is not an isolated phenomenon but is embedded within a broader set of anthropogenic pressures that influence microbial ecosystems at the planetary scale. Land-use change, chemical contamination, climate-driven stress, and global connectivity interact to reshape microbial communities and selection dynamics. Within this framework, microplastics represent an additional layer of environmental complexity that can amplify existing drivers of antimicrobial resistance. Micro- and nanoplastics provide surfaces for microbial colonization, giving rise to dense biofilm communities known as the plastisphere. Rather than an isolated hotspot, the plastisphere can be understood as a distributed network of microhabitats embedded within larger environmental systems, where local processes can scale up to influence global resistance dynamics (1). These biofilms constitute highly interactive ecological systems in which microorganisms, pollutants, and genetic material converge. Plastics can accumulate antibiotics, heavy metals, and other contaminants, thereby generating localized selective pressures that favor resistant organisms (2). Beyond serving as substrates for microbial growth, plastisphere communities may function as evolutionary interfaces that reshape microbial adaptation, gene flow, and resistome dynamics across ecosystems. The plastisphere is therefore not simply a reservoir of resistant bacteria, but a dynamic environment that may accelerate resistance evolution and dissemination (Fig. 1).

Emerging evidence indicates that the influence of plastics on antimicrobial resistance is strongly linked to environmental complexity. Increasing microplastic diversity, including variation in polymer type, shape, and physicochemical properties, enhances the abundance of resistance genes, mobile genetic elements, and virulence factors in microbial communities. These findings show that heterogeneous plastic pollution acts as a composite ecological driver, amplifying both selection and diversification within microbial populations (3, 4).

Micro- and nanoplastics can directly alter microbial physiology and gene exchange dynamics. Exposure to these particles induces oxidative stress, increases membrane permeability, and activates stress response pathways that facilitate horizontal gene transfer (5–7). Experimental evidence shows that even environmentally relevant concentrations of micro- and nanoplastics can enhance the conjugative transfer of resistance genes, including between different taxa (5, 7). These responses involve increased intracellular energy availability and structural changes that promote close cell-to-cell contact, enabling efficient genetic exchange under environmentally relevant conditions. Together, these processes show that plastics not only impose selective pressure but also directly influence the mechanisms underlying microbial adaptation and gene flow.

These effects depend strongly on the physicochemical properties of plastic particles. Particle size, in particular, is a key determinant of microbial dynamics. Nanoplastics, owing to their high surface area and reactivity, intensify microbial contact and promote dense gene transfer networks. Smaller particles increase the colocalization of resistance genes with mobile genetic elements and enhance the frequency of horizontal gene transfer, thereby transforming plastisphere communities into active hubs of genetic exchange (7). From an evolutionary perspective, these environments increase mutation supply, selection intensity, and gene flow simultaneously, creating conditions that favor rapid adaptation and the stabilization of resistance traits.

Microplastics also interact with other environmental stressors rather than acting alone. Antibiotics, heavy metals, and biocides can coaccumulate on plastic surfaces, while environmental fluctuations such as temperature and hydrological variability further influence microbial activity and gene transfer. These combined pressures create conditions that intensify selection and accelerate the spread of antimicrobial resistance beyond the effects of individual drivers. Although direct quantification of plastisphere-mediated transfer into clinical pathogens remains limited, accumulating evidence suggests that these processes may increase ecological connectivity between environmental resistomes and clinically relevant bacteria (8).

Within the plastisphere, complex interactions emerge between mobile genetic elements and microbial defense systems that define the resistome. Plasmids, transposons, and bacteriophages act as key vehicles for gene dissemination, while bacterial defense mechanisms regulate gene flow. Evidence indicates that these components can coexist and coevolve, contributing both to genetic exchange and to genomic stability. Resistance evolution in the plastisphere is therefore governed by a balance between mobility and control, rather than by gene transfer alone (9).

A striking aspect of this emerging framework is the role of biodegradable plastics, which are widely promoted as environmentally friendly alternatives. However, these materials can create nutrient-rich and high-stress microenvironments that favor microbial growth and enhance gene transfer processes. Studies show that biodegradable plastispheres can exhibit higher resistome risk, increased abundance of mobile genetic elements, and enhanced dissemination of resistance genes compared to conventional plastics, reflecting intensified interactions between mobile genetic elements and microbial defense systems in biodegradable plastispheres (6, 9). These findings challenge the assumption that biodegradable plastics reduce environmental risk and point to the need for a more comprehensive evaluation of their ecological impact.

A key challenge is translating these processes from controlled experiments to natural environments. Plastics move between environmental compartments, connecting aquatic, terrestrial, and atmospheric systems. This mobility suggests that plastisphere-mediated resistance dynamics operate across spatial scales, linking local microbial interactions with broader dissemination pathways. As a result, plastic-associated microbial processes may contribute to the spread of resistance beyond their immediate environments.

Despite increasing evidence, important knowledge gaps remain. There is limited understanding of how plastic diversity, particle size, and environmental aging interact to determine resistance selection thresholds, and the contribution of plastisphere-mediated gene transfer to clinically relevant resistance pathways remains poorly quantified. Current monitoring frameworks do not incorporate the role of plastics in shaping environmental resistomes, which limits their integration into One Health approaches.

The implications for policy and risk assessment are substantial. Plastic pollution is increasingly recognized as both an environmental issue and a public health concern, with growing calls for immediate action even in the absence of a binding global agreement (10). However, existing regulatory frameworks largely overlook its biological and evolutionary dimensions. Integrating resistome dynamics, gene mobility, and microbial interactions into plastic risk assessment is essential to better align environmental policies with antimicrobial resistance emergence.

Recognizing the plastisphere as an evolutionary hotspot reframes our understanding of plastic pollution. Micro- and nanoplastics act not only as contaminants but also as drivers of microbial adaptation, facilitating the emergence and dissemination of antimicrobial resistance. Integrating environmental science, microbiology, and policy will be critical to anticipate and mitigate these risks. Plastic pollution should therefore be understood as both an environmental challenge and a driver of evolutionary change with far-reaching consequences for microbial ecosystems and global health.
